# Tonsillectomy Does Not Reduce Upper Respiratory Infections: A National Cohort Study

**DOI:** 10.1371/journal.pone.0169264

**Published:** 2016-12-30

**Authors:** Hyo Geun Choi, Bumjung Park, Songyong Sim, Soon-Hyun Ahn

**Affiliations:** 1 Department of Otorhinolaryngology-Head & Neck Surgery, Hallym University College of Medicine, Anyang, Korea; 2 Department of Statistics, Hallym University, Chuncheon, Korea; 3 Department of Otorhinolaryngology-Head & Neck Surgery, Seoul National University College of Medicine, Bundang, Korea; University of Insubria, ITALY

## Abstract

**Objectives:**

The objective of this study was to compare post-operative visits for upper respiratory infections (URIs) between tonsillectomy and non-tonsillectomy participants (controls).

**Methods:**

Using the national cohort study from the Korean Health Insurance Review and Assessment Service, 1:4 matched (age, sex, income, region, and pre-operative URI visit) tonsillectomy participants (5,831) and control participants (23,324) were selected. Post-operative visits for URI were measured from 1 to 9 years post-op. The equivalence test was used. The margin of equivalence of the difference (Tonsillectomy—Control group group) was set to -0.5 to 0.5.

**Results:**

There was no difference between the tonsillectomy and control group in 1- to 9-year post-op visits (-0.5 < 95% CI of difference < 0.5). URI visits gradually decreased from 5.5/2 years (pre-op) to 2.1/year (at 1 year post-op) and 1.4/year (at 9 years post-op) in both tonsillectomy and control groups. In the subgroup analysis (children Vs adolescent and adults; rare Vs frequent pre-operative URI), there was no difference in the number of post-op visits for URI between the tonsillectomy and control groups (-0.5 < 95% CI of difference < 0.5).

**Conclusion:**

Tonsillectomy does not provide a decrease in the number of post-operative visits for URI, and URI decreased over time whether or not a tonsillectomy was performed.

## Introduction

Tonsillectomy with/without adenoidectomy is one of the most commonly performed surgeries, especially in children [1–3]. The reported rate of tonsillectomy in children and adolescents is 7.9 per 1,000 in the US [4] and 2.6 per 1,000 in Korea [3]. Tonsillectomy is generally performed for recurrent sore throat or obstructive sleep apnea (OSA) [5, 6]. However, there are no nationally accepted guidelines for when to perform a tonsillectomy [7]. Although Paradise et al reported the “paradise criteria" for tonsillectomy in recurrent tonsillitis [2, 8], the evidence for its clinical efficacy is limited [9]. Some authors have reported that tonsillectomy reduced the incidence of pediatric URI [8, 10], while others have reported that it did not [11]. Even with the current lack of robust clinical evidence, infection (23.2%) remains one of the most common reasons for performing a tonsillectomy [12].

Tonsillectomies are associated with the possibility of complications. Late postoperative bleeding, which has a rate of 1–5%, is the most common complication [7]. Infrequent complications, such as early postoperative bleeding, taste disorder, nasal speech, vascular injury, emphysema, and dysphagia, have also been reported [13]. We should therefore carefully consider whether tonsillectomy should be performed for recurrent sore throat.

The purpose of this study is to compare post-operative visits for upper respiratory infection (URI) between tonsillectomy and non-tonsillectomy participants (controls) using a national cohort study. In this study, we matched the tonsillectomy and control group at 1:4 for age, sex, income group, and the number of pre-operative URIs. We followed up the participants for 1 to 9 years.

## Materials and Methods

### Study Population and Data Collection

The ethics committee of Hallym University (2014-I148) approved the use of these data. Written informed consent was exempted by the Institutional Review Board.

This national cohort study relies on data from the Korean Health Insurance Review and Assessment Service—National Patient Sample (HIRA-NPS). The Korean National Health Insurance Service (NHIS) selects samples directly from the entire population database to prevent non-sampling errors. Approximately 2% of the samples (one million) were selected from the entire Korean population (50 million). This selected data can be classified at 1,476 levels (age [18 categories], sex [2 categories], and income level [41 categories]) using randomized stratified systematic sampling methods via proportional allocation to represent the entire population. After data selection, the appropriateness of the sample was verified by a statistician who compared the data from the entire Korean population to the sample data. The details of the methods used to perform these procedures are provided by the National Health Insurance Sharing Service [14]. This cohort database included (i) personal information, (ii) health insurance claim codes (procedures and prescriptions), (iii) diagnostic codes using the International Classification of Disease-10 (ICD-10), (iv) socio-economic data (residence and income), and (v) medical examination data for each participant over a period ranging from 2002 to 2013.

Because all Korean citizens are recognized by a 13-digit resident registration number from birth to death, exact population statistics can be determined using this database. It is mandatory for all Koreans to enroll in the NHIS. All Korean hospitals and clinics use the 13-digit resident registration number to register individual patients in the medical insurance system. Therefore, the risk of overlapping medical records is minimal, even if a patient moves from one place to another. Moreover, all medical treatments in Korea can be tracked without exception using the HIRA system.

### Participants Selection

**Fig 1:**
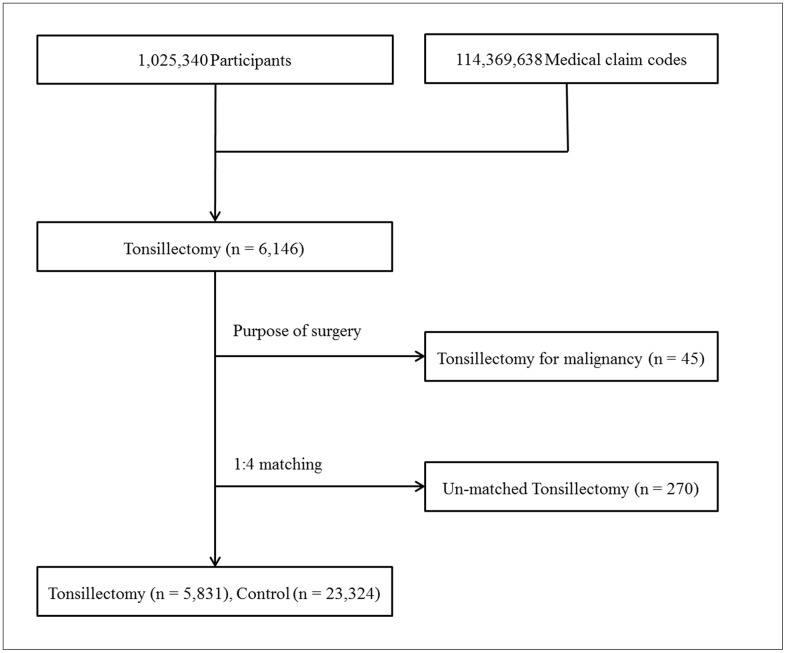
A schematic illustration of the participant selection process that was used in the present study. Out of a total of 1,025,340 participants, 6,146 tonsillectomy participants were selected. Tonsillectomy for malignancy was excluded (n = 45). The tonsillectomy participants were matched 1:4 with a control group that did not undergo tonsillectomy. Un-matched tonsillectomy participants were excluded (n = 270). Finally, 5,831 tonsillectomy participants and 23,324 control participants were included.

Out of 1,025,340 cases with 114,369,638 medical claim codes, we included participants who underwent tonsillectomy (claim code: Q2300) from 2004 through 2012 (n = 6,146). Among these, participants who underwent tonsillectomy for malignancies were excluded (n = 45). Hence, only participants who underwent tonsillectomy for benign causes (e.g., chronic tonsillitis, chronic tonsillar hypertrophy, and obstructive sleep apnea) were included. The tonsillectomy participants were matched 1:4 with the participants (control group) who never underwent tonsillectomy from 2002 through 2013 among this cohort. The matches were processed for age, group, sex, income group, region of residence, and the number of pre-operative URI histories over 2 years. To prevent selection bias when selecting the matched participants, the control group participants were sorted using a random number order, and they were then selected from top to bottom. The tonsillectomy participants for whom we could not identify enough matching participants were excluded (n = 270). Finally, 1:4 matching resulted in the inclusion of 5,831 of tonsillectomy participants and 23,324 control participants (Fig 1).

### Variables

The age groups were classified using 5-year intervals: 0–4, 5–9, 10–15…, and 80+ years old. A total of 17 age groups were designated. The income groups were initially divided into 41 classes (one health aid class, 20 self-employment health insurance classes, and 20 employment health insurance classes). These groups were re-categorized into 11 classes (class 1 [lowest income]-11 [highest income]). Region of residence was divided into 16 areas according to administrative district. These regions were regrouped into urban (Seoul, Busan, Daegu, Incheon, Gwangju, Daejeon, and Ulsan) and rural (Gyeonggi, Gangwon, Chungcheongbuk, Chungcheongnam, Jeollabuk, Jeollanam, Gyeongsangbuk, Gyeongsangnam, and Jeju) areas.

**Fig 2:**
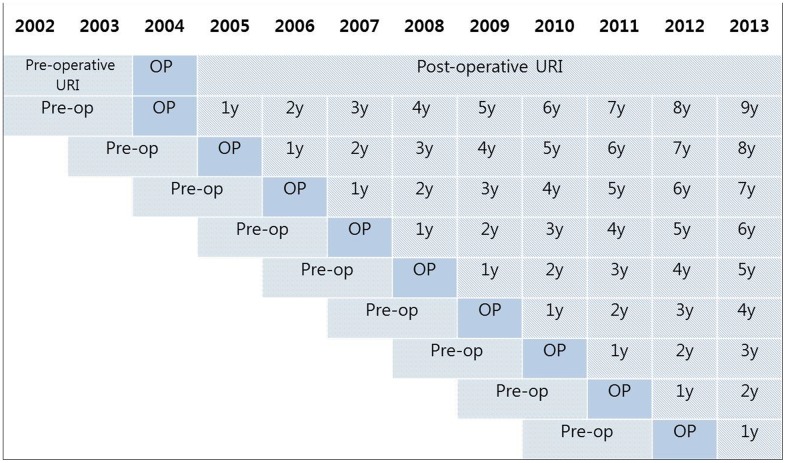
A schematic illustration of the measurements used for preoperative and postoperative URI visits. The number of visits for URI was measured during the 2 years before tonsillectomy. Post-operative visits for URI were counted for 1 to 9 years, depending on the time of the surgery.

We defined URI using the following ICD-10 codes: J00 (acute nasopharyngitis) and J02 (acute pharyngitis) through J069 (acute upper respiratory infection). The number of visits to clinics or hospital for URI was counted every year. Pre-operative URI visits were counted for 2 years. The number of visits included in the URI history during the follow up period was counted for each year (e.g., post-op year 1, year 2, year 3…. year 9). Therefore, the participants who underwent tonsillectomy in 2004 were followed up for 9 years, while the participants who underwent tonsillectomy in 2012 were followed up for 1 year (Fig 2).

### Statistical Analyses

An equivalence test was used to compare the number of visits for URI (pre-operative and in post-op year 1, year 2, year 3 … year 9) between the tonsillectomy group and the control group. The null hypothesis was that visits for URI during the follow up period would not be the same between the tonsillectomy and control groups. In a previous meta-analysis, the pooled risk difference in URIs between tonsillectomy and control groups was -0.5 episodes per year.[9] Therefore, the margin of equivalence of difference (tonsillectomy—control group group) was set to -0.5 to 0.5 in this study.

For the subgroup analyses, the participants were divided into 2 groups: children (≤ 14 years old) and adolescent and adults (≥ 15 years old); and rare pre-operative URI (< 5 times over 2 years) and frequent pre-operative URI (≥ 5 times over 2 years).

For the equivalence test, a 95% confidence interval (CI) for a difference < 0.5 was considered to indicate statistical significance. The results were statistically analyzed using SPSS v. 21.0 (IBM, Armonk, NY, USA).

## Results

Because the patients were matched, the general characteristics (e.g., age group, sex, and income level) were the same in both groups (Table 1). The number of visits for pre-operative URI was also exactly the same in both groups (Table 2).

**Table 1:** General Characteristics of Participants.

Characteristics	The Number of participants (matched 1:4)		
	Tonsillectomy group	Control group	Total participants
Age (years old)			
0–4	1,661	6,644	8,305
5–9	1,089	4,356	5,445
10–14	692	2,768	3,460
15–19	521	2,084	2,605
20–24	460	1,840	2,300
25–29	412	1,648	2,060
30–34	331	1,324	1,655
35–39	225	900	1,125
40–44	184	736	920
45–49	124	496	620
50–54	56	224	280
55–59	44	176	220
60–64	27	108	135
65–69	4	16	20
70–74	0	0	0
75–79	1	4	5
Sex			
Male	3,432	13,728	17,160
Female	2,399	9,596	11,995
Income			
1 (lowest)	50	200	250
2	215	860	1,075
3	270	1,080	1,350
4	365	1,460	1,825
5	495	1,980	2,475
6	569	2,276	2,845
7	669	2,676	3,345
8	790	3,160	3,950
9	835	3,340	4,175
10	838	3,352	4,190
11 (highest)	735	2,940	3,675
Region of residence			
Urban	2,634	10,536	13,170
Rural	3,197	12,788	15,985

**Table 2:** Differences in mean values for pre-operative and post-operative URIs between the tonsillectomy and control groups.

	Tonsillectomy (mean, SD)	Control (mean, SD)	95% CI of the difference	P-value
Pre-op URI for 2 y (n = 29,154)	5.5 ± 5.3	5.5 ± 5.3	-0.2 to 0.2	1.000
Post-op 1 y URI (n = 29,154)	2.1 ± 3.0	2.0 ± 3.0	0.0 to 0.2	0.022
Post-op 2 y URI (n = 26,915)	2.1 ± 3.1	1.9 ± 3.0	0.0 to 0.2	0.027
Post-op 3 y URI (n = 24,180)	2.0 ± 3.0	1.9 ± 3.0	0.0 to 0.2	0.252
Post-op 4 y URI (n = 21,840)	1.9 ± 3.0	1.8 ± 3.1	-0.1 to 0.1	0.618
Post-op 5 y URI (n = 18,825)	1.8 ± 2.9	1.7 ± 2.8	0.0 to 0.2	0.087
Post-op 6 y URI (n = 15,340)	1.7 ± 2.7	1.6 ± 2.7	-0.1 to 0.2	0.350
Post-op 7 y URI (n = 10,605)	1.5 ± 2.4	1.5 ± 2.5	-0.1 to 0.1	0.651
Post-op 8 y URI (n = 7,505)	1.4 ± 2.4	1.4 ± 2.5	-0.1 to 0.2	0.667
Post-op 9 y URI (n = 3,850)	1.4 ± 2.3	1.3 ± 2.3	-0.1 to 0.3	0.365

URI: Upper respiratory infection

SD: Standard deviation

Difference: Tonsillectomy group—Control group

CI: Confidence interval

We compared the visits for URI during the follow up period. There was no difference between the tonsillectomy and control groups from post-op year 1 year through year 9 (-0.5 < 95% CI of difference < 0.5). We found that URI visits gradually decreased from 5.5/2 year (pre-op) to 2.1/year (in post-op year 1), 2.1/year (post-op year 2), 2.0/year (post-op year 3), 1.9/year (post-op year 4), 1.8/year (post-op year 5), 1.7/year (post-op year 6), 1.5/year (post-op year 7), 1.4/year (post-op year 8), and 1.4/year (post-op year 9) in the tonsillectomy group. The same changes were also observed in the control group (Table 2).

In the subgroup analysis (children Vs adolescent and adults; and rare Vs frequent pre-operative URI), there was no difference in the number of visits for URI between the tonsillectomy and control groups (-0.5 < 95% CI of difference < 0.5) during the follow up periods from 1 to 9 years post-op. The ‘children’ and ‘frequent pre-operative URI’ groups showed a stronger decrease in the number of visits for URI during the follow up periods than the ‘adolescent and adults’ and ‘rare pre-operative URI’ groups (Table 3). We added the difference of URIs between tonsillectomy and control groups in the very frequent pre-operative URI group (≥ 6 times a year, S1 Table).

**Table 3:** Subgroup analysis of mean values for pre-operative and post-operative URIs between the tonsillectomy and control groups (children Vs adolescents and adults; and rare Vs frequent pre-operative URIs).

	Tonsillectomy (mean, SD)	Control (mean, SD)	95% CI of difference	P-value
**Children (≤ 14 years old)**				
Pre-op URI for 2 y (n = 17,210)	7.0 ± 5.7	7.0 ± 5.7	-0.2 to 0.2	1.000
Post-op 1 y URI (n = 17,210)	2.5 ± 3.2	2.5 ± 3.3	-0.1 to 0.2	0.320
Post-op 2 y URI (n = 16,050)	2.4 ± 3.4	2.3 ± 3.4	-0.1 to 0.2	0.437
Post-op 3 y URI (n = 14,615)	2.2 ± 3.2	2.2 ± 3.3	-0.2 to 0.1	0.723
**Adolescents and adults (≥ 15 years old)**				
Pre-op URI for 2 y (n = 11,945)	3.3 ± 3.5	3.3 ± 3.5	-0.2 to 0.2	1.000
Post-op 1 y URI (n = 11,945)	1.6 ± 2.5	1.4 ± 2.4	0.0 to 0.3	0.005
Post-op 2 y URI (n = 10,865)	1.6 ± 2.7	1.4 ± 2.4	0.1 to 0.3	0.004
Post-op 3 y URI (n = 9,565)	1.6 ± 2.6	1.5 ± 2.6	0.0 to 0.4	0.008
**Rare pre-operative URI (< 5 times for 2 y)**				
Pre-op URI for 2 y (n = 15,725)	1.7 ± 1.4	1.7 ± 1.4	-0.1 to 0.1	1.000
Post-op 1 y URI (n = 15,725)	1.3 ± 2.1	1.0 ± 1.8	0.2 to 0.3	< 0.001
Post-op 2 y URI (n = 14,280)	1.3 ± 2.2	1.0 ± 1.9	0.2 to 0.3	< 0.001
Post-op 3 y URI (n = 12,700)	1.3 ± 2.2	1.1 ± 2.0	0.1 to 0.3	< 0.001
**Frequent pre-operative URI (≥ 5 times for 2 y)**				
Pre-op URI for 2 y (n = 13,430)	9.9 ± 4.7	9.9 ± 4.7	-0.2 to 0.2	1.000
Post-op 1 y URI (n = 13,430)	3.1 ± 3.5	3.2 ± 3.7	-0.2 to 0.1	0.362
Post-op 2 y URI (n = 12,635)	2.9 ± 3.7	3.0 ± 3.7	-0.2 to 0.1	0.442
Post-op 3 y URI (n = 11,480)	2.7 ± 3.4	2.8 ± 3.7	-0.3 to 0.1	0.206

URI: Upper respiratory infection

SD: Standard deviation

Difference: Tonsillectomy group—Control group

CI: Confidence interval

## Discussion

We found no difference in the number of URI visits between the tonsillectomy and control groups. After tonsillectomy, URI visits gradually decreased during the follow up period. However, this trend was also observed in control group, which showed the same scale.

According to the most recent Cochrane library review [15], which reviewed 7 randomized controlled trials (RCTs) that included children and 2 RCTs that included adults, the number of post-op 1 year episodes of sore throat was lower in the tonsillectomy group than in the control group, and this difference was not observed after post-op year 2 year in children. They also reported that the size of the effect (e.g., tonsillectomy for chronic/acute tonsillitis in children) was very modest because some of the children improved without surgery [16]. Another review study concluded that the frequencies of sore throat episodes and upper respiratory infections became lower over time whether or not a tonsillectomy was performed [9]. Our study supports these previous results. However, we found no difference between the tonsillectomy and control groups even during post-op year 1. We think that this might be because of the relatively low incidence of pre-operative URI visits in this study population (5.5 visits over 2 years). In previous studies, the effects of tonsillectomy were not evident in mild symptoms groups, whereas effects were evident in moderate to severe symptoms groups (3–6 throat infections) [11].

Historically, the tonsils have been regarded as sources of bacteria [17]. Therefore, tonsillectomy was performed to remove an apparent infection focus in recurrent sore throat patients [17]. However, bacterial pathogens have since been discovered not only children and adults with recurrent tonsillitis but also in healthy children and adults [18]. Moreover, asymptomatic carriers (10% of all healthy children) of staphylococci and streptococci do not require treatment [7]. Bacterial pathogens that reside in the tonsils do not always provoke problems in healthy individuals. Therefore, we believe that removing the tonsils is not an effective way to treat patients who present with mild recurrent sore throat. As children grow up, their immune systems come into maturity. This might be responsible for decreases that have been observed in the number of URIs reported during follow up periods between individuals who did or did not undergo tonsillectomy [7, 9]. In this study, URI was not decreased or increased after tonsillectomy compared to control. It means that tonsillectomy would not increase the possibility of infection by affecting immune system.

One advantage of this study is the large number of study participants (n = 29,154). We followed up the tonsillectomy group for a maximum of 9 years, whereas other studies have usually used a 2-year follow up [2, 8, 11, 19–22]. To our knowledge, this is the largest study to evaluate the efficacy of tonsillectomy for URI. Another advantage is the availability of comprehensive medical records for each participant. Previous studies needed to ask the participants for their histories of recurrent sore throat [2, 8, 11, 19–22], and this could result in recall bias. In this study, we used patient medical records and HIRA data to count URI visits. These recorded data are not distorted by patient memory. The HIRA data include all citizens of the nation, without exception. We therefore were not missing any participants during the follow up periods, whereas a significant loss to follow up has been a problem in other studies [2, 8]. In our control group, none of the participants changed during observation to the tonsillectomy group. However, we did not use RCT methods, but instead exactly matched our participants to individuals in the control group according to age, sex, income, region of residence, and previous operative URI visits. Income and region matching were important because these are determinant factors of medical procedures. Income levels can be determined very accurately using the Korean NHIS because a patient’s premium is determined based on their income. Our study results are therefore representative of the entire Korean population because the data were selected from a database that covers the entire population, and the representativeness of the data was verified by a statistician.

Our study has several limitations. We used health insurance claims data and counted the number of visits for URI. These do not exactly reflect the number of infections. However, we believe that the number of visits for URI can be used as a surrogate index for the number of infections when dealing with this type of big data. We could not measure the severity of each URI in each participant. The medical procedures used for each participant could be variable even when treating the same disease. We included participants who underwent tonsillectomy for chronic tonsillar hypertrophy or OSA because we could not determine the purpose of tonsillectomy in each participant. However, if tonsillectomy reduces URI, the number of URIs in the participants who underwent tonsillectomy for chronic tonsillar hypertrophy or OSA should also be decreased.

## Conclusion

Tonsillectomy does not provide a benefit against URIs. It does appears that tonsillectomy decreases URIs, but URIs also decreased over time whether or not a tonsillectomy was performed.

## Supporting Information

S1 Table Subgroup analysis of mean values for pre-operative and post-operative URIs between the tonsillectomy and control groups in very frequent pre-operative URI group (≥ 12 times for 2 y). (DOCX)

## Abbreviations

CI: confidence interval
HIRA: Korean Health Insurance Review and Assessment Service
NHIS: The Korean National Health Insurance Service
RCT: randomized controlled trial
URI: upper respiratory infection
