# Supplementary material for: Tonsillectomy Does Not Reduce Upper Respiratory Infections: A National Cohort Study
Source: PLoS One. 2016 Dec 30;11(12):e0169264. doi: 10.1371/journal.pone.0169264 (PMC5201261; doi:10.1371/journal.pone.0169264)
Supplement: S1 Table — (DOCX) [file pone.0169264.s001.docx]

S1 Table Subgroup analysis of mean values for pre-operative and post-operative URIs between the tonsillectomy and control groups in very frequent pre-operative URI group (≥ 12 times for 2 y).

|  | Tonsillectomy  (mean, SD) | Control  (mean, SD) | 95% CI of difference | P-value |
| --- | --- | --- | --- | --- |
| **Frequent pre-operative URI**  **(≥ 12 times for 2 y)** |  |  |  |  |
| Pre-op URI for 2 y (n = 3,905) | 15.9 ± 3.8 | 15.9 ± 3.8 | -0.3 to 0.3 | 1.000 |
| Post-op 1 y URI (n = 3,905) | 4.5 ± 4.1 | 4.7 ± 4.5 | -0.5 to 0.1 | 0.245 |
| Post-op 2 y URI (n = 3,740) | 4.0 ± 4.6 | 4.2 ± 4.6 | -0.5 to 0.2 | 0.329 |
| Post-op 3 y URI (n = 3,465) | 3.6 ± 4.1 | 3.9 ± 4.4 | -0.7 to 0.0 | 0.060 |

URI: Upper respiratory infection

SD: Standard deviation

Difference: Tonsillectomy group - Control group

CI: Confidence interval
